# Supplementary figures and images for: Effects of intention understanding and brief imitative experience on the mirror neuron system: An EEG study using Japanese sign language
Source: PLoS One. 2025 Dec 19;20(12):e0335885. doi: 10.1371/journal.pone.0335885 (PMC12716736; doi:10.1371/journal.pone.0335885)

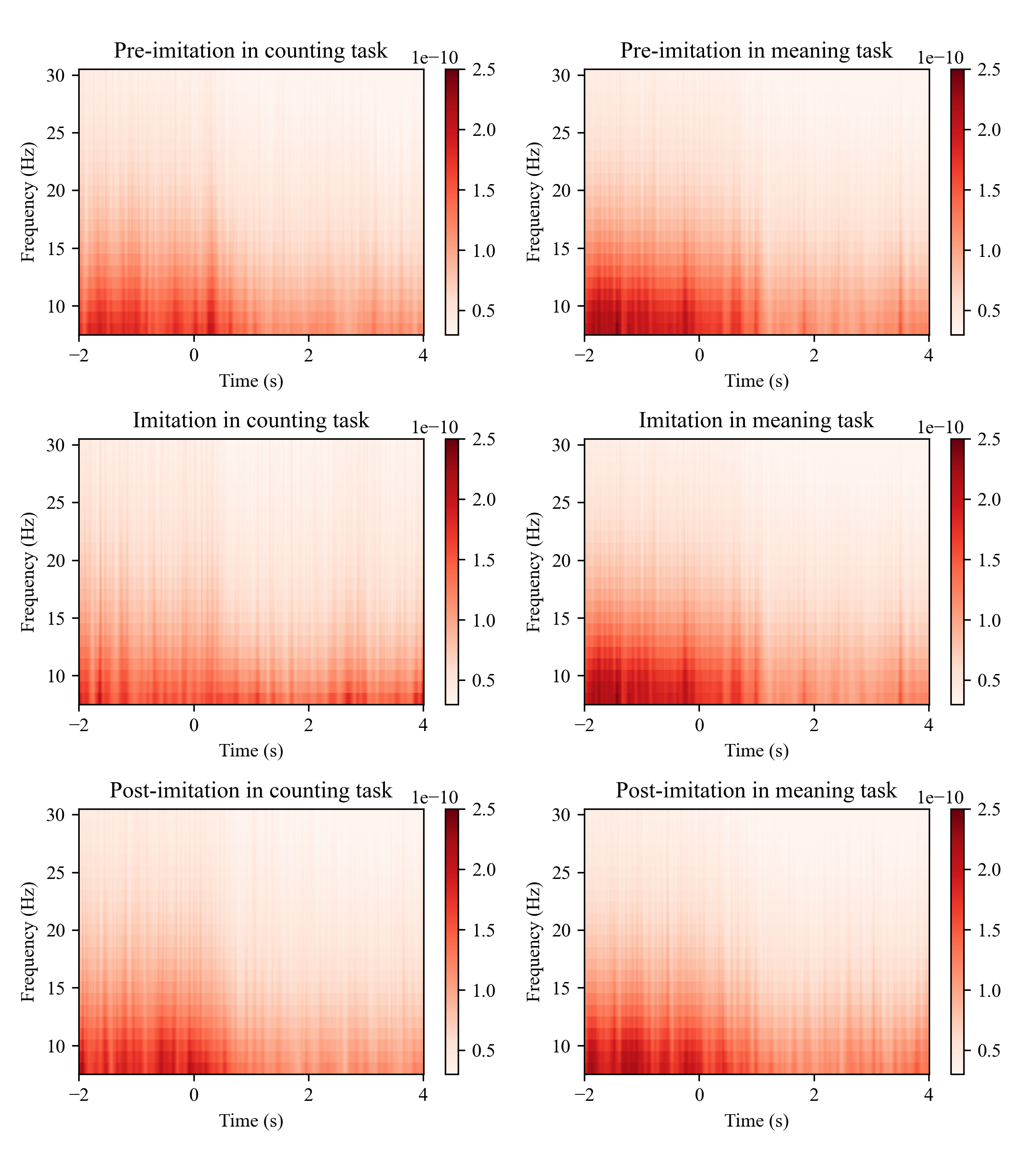

Supplement: S1 Fig — The left columns show time-frequency plots in the counting task and the right ones show the plots in the meaning task (Top: pre-imitation, Middle: imitation, Bottom: post-imitation). (TIF) [file pone.0335885.s002.tif]

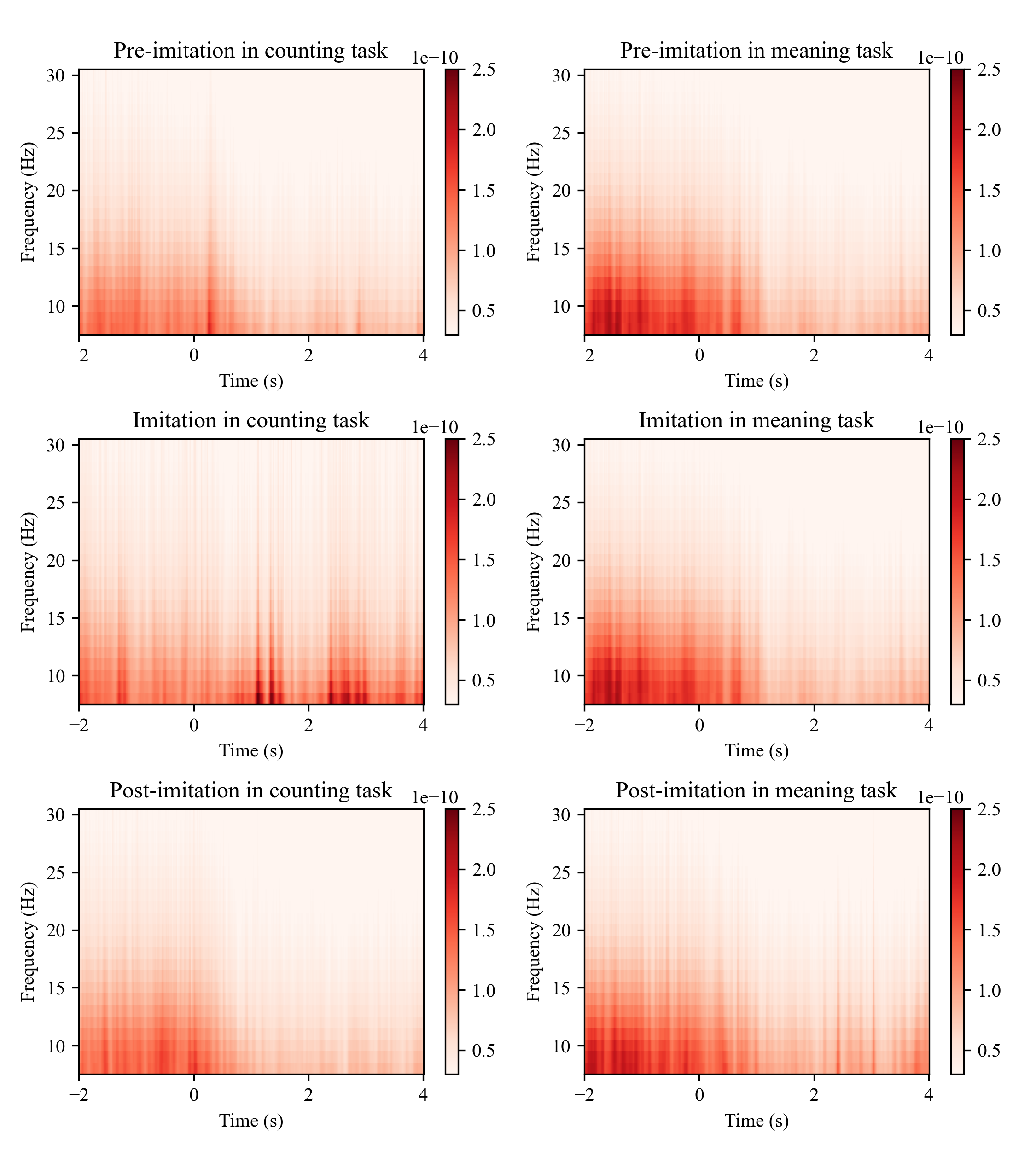

Supplement: S2 Fig — The layout of the figures are identical to S1 Fig. (TIF) [file pone.0335885.s003.tif]
