# Supplementary material for: Effects of intention understanding and brief imitative experience on the mirror neuron system: An EEG study using Japanese sign language
Source: PLoS One. 2025 Dec 19;20(12):e0335885. doi: 10.1371/journal.pone.0335885 (PMC12716736; doi:10.1371/journal.pone.0335885)
Supplement: Attachment — Submitted filename: Response_to_Reviewers_auresp_2.docx [file pone.0335885.s005.docx]

Dear Reviewer1,

We are mostly grateful for additional suggestions that will further improve our manuscript. We seriously took your suggestions into consideration, and reflected them on the revised manuscript. We hope the revised manuscript successfully addressed your concerns.

**Comment 1**

Is this a typo? Line 381: “Each orage dot”

**Response**

Appreciating the reviewer’s comment, we fixed the typo on pp.17.

**Comment 2**

Results of machine learning: besides accuracy, it might be interesting to report other performance metrics.

**Response**

Following the plausible suggestion, we added precision, recall, and F1-score of the target and randomized machines on pp.24-25. We also included the results of permutation tests and their p-values.

**Comment 3**

Line 710: I would rephrase this sentence without explicitly mentioning the statistical test: “BANOVA favored null hypotheses as for main effects of the task and imitative experience as …”

**Response**

We followed the reviewer's comment and rephrased the sentence as below.

“While beta suppression in the central regions may correspond to excitation of the MNS, the suppression was not modulated by the task type, imitative experience, and their interaction.” (pp.32).

**Comment 4**

Conclusions (Lines 737–741): I would rephrase the final sentence in a more cautious manner, also considering that, when discussing autism, different levels of severity exist. Moreover, the exact neurobiological mechanisms underlying this condition are not yet known.

**Response**

As the reviewer suggested, autism is characterized by multidimensional variation, and its neurobiological underlying is less known. Because the present study was not directly related to autism, we decided to delete the sentences addressing the disorder in conclusion (pp.33)

**Comment 5**

Line 744: if the acknowledgments section has been removed, the corresponding heading should also be deleted.

**Response**

Following the reviewer’s comment, we removed the heading of acknowledgement on pp.34.
